# Calcium-Dependent S100A8 Amyloid Fibril Formation via S100A1-Mediated Transient Interaction

**DOI:** 10.1021/acschemneuro.5c00086

**Published:** 2025-06-25

**Authors:** Viktorija Karalkevičiu̅tė, Ieva Baronaitė, Aistė Peštenytė, Dominykas Veiveris, Gediminas Usevičius, Mantas Šimėnas, Mantas Žiaunys, Vytautas Smirnovas, Darius Šulskis

**Affiliations:** † Institute of Biotechnology, Life Sciences Center, 54694 Vilnius University, LT-10257 Vilnius, Lithuania; ‡ Faculty of Physics, Vilnius University, LT-10222 Vilnius, Lithuania

**Keywords:** amyloid, neurodegeneration, inflammation, aggregation, S100, interaction

## Abstract

The S100 family consists of calcium-binding proteins that are largely known for their contribution to neuroinflammatory processes. These proteins are associated with various cardiac and neurological functions as well as related diseases. A few S100 proteins can form unspecific or amyloid aggregates in neuropathologies and thus play a part in dementia pathogenesis. Among all S100 proteins, S100B and S100A9 aggregation properties are the most investigated; however, there is a lack of studies regarding other S100 members. In particular, S100A1 and S100A8 are also associated with neurological pathologies, but their interactions and aggregation are poorly understood. Therefore, in this study, we explored whether S100A1 and S100A8 proteins can form heterodimers, interact, or coaggregate. Our results revealed that S100A1 and S100A8 interactions and S100A8 amyloid aggregation are driven by calcium ions. We observed that while S100A1 remains mostly stable, S100A8 forms various types of spherical or unspecific aggregates. While they do not form stable heterodimers like calprotectin, their transient interactions facilitate the formation of worm-like amyloid fibrils, and the process is regulated by different calcium ion concentrations. At calcium ion saturation, both proteins are stabilized, leading to inhibition of aggregation. Overall, by employing a diverse range of techniques from amyloid and protein-specific fluorescence detection to electron–electron double resonance spectroscopy, we elucidated interactions between S100 proteins that might otherwise be overlooked, enhancing our understanding of their aggregation behavior.

## Graphical abstract

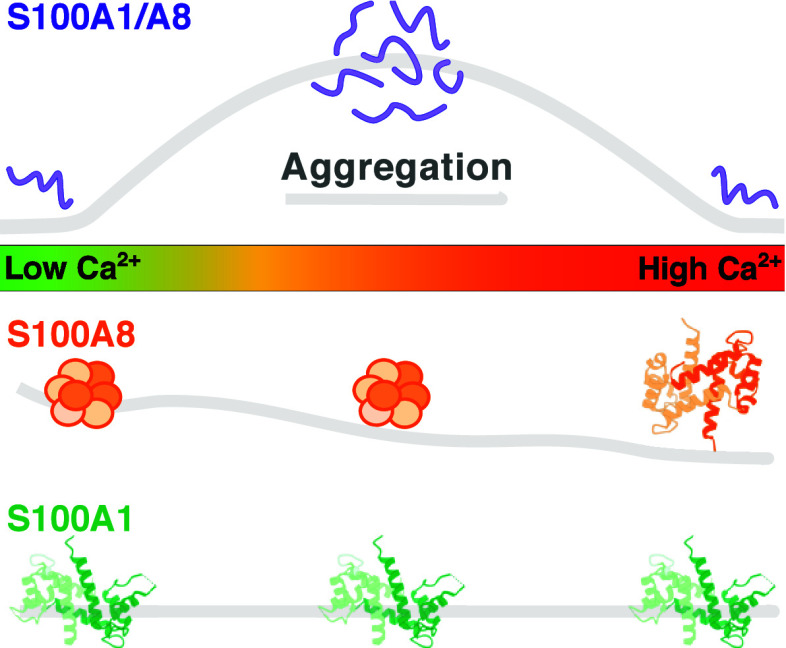

## Introduction

The S100 is a calcium-binding protein family with at least 21 members found in various tissues.^1^ These members have numerous intracellular and extracellular functions that range from apoptosis, inflammation, and homeostasis to regulating other cells.^2^ S100 proteins are found within cells mainly as homodimers, with some key members forming heterodimers for specific functions.^3^ The conformation of S100 is controlled by two EF-hand structural motifs that bind calcium ions and are essential for S100 functions.^4^ Furthermore, the majority of their interactions with their clients are calcium-dependent, allowing them to dynamically participate in calcium signaling pathways.^1^ Contributing to that, there is an established connection between calcium ions and S100 oligomerization, as they stabilize dimers but can also assist in assembling larger oligomers or heterodimers.^5^ S100 proteins are located in different parts of the body;^2^ however, historically they were first discovered in the brain.^6^ Currently, seven members (S100B, S100A1, S100A6, S100A7, S100A8, S100A9, and S100A12) are known to localize within the brain and are associated with Alzheimer’s disease,^7^ regulation of neuroinflammation, and neuroactivation^8^ (Figure 1A).

**Figure 1:**
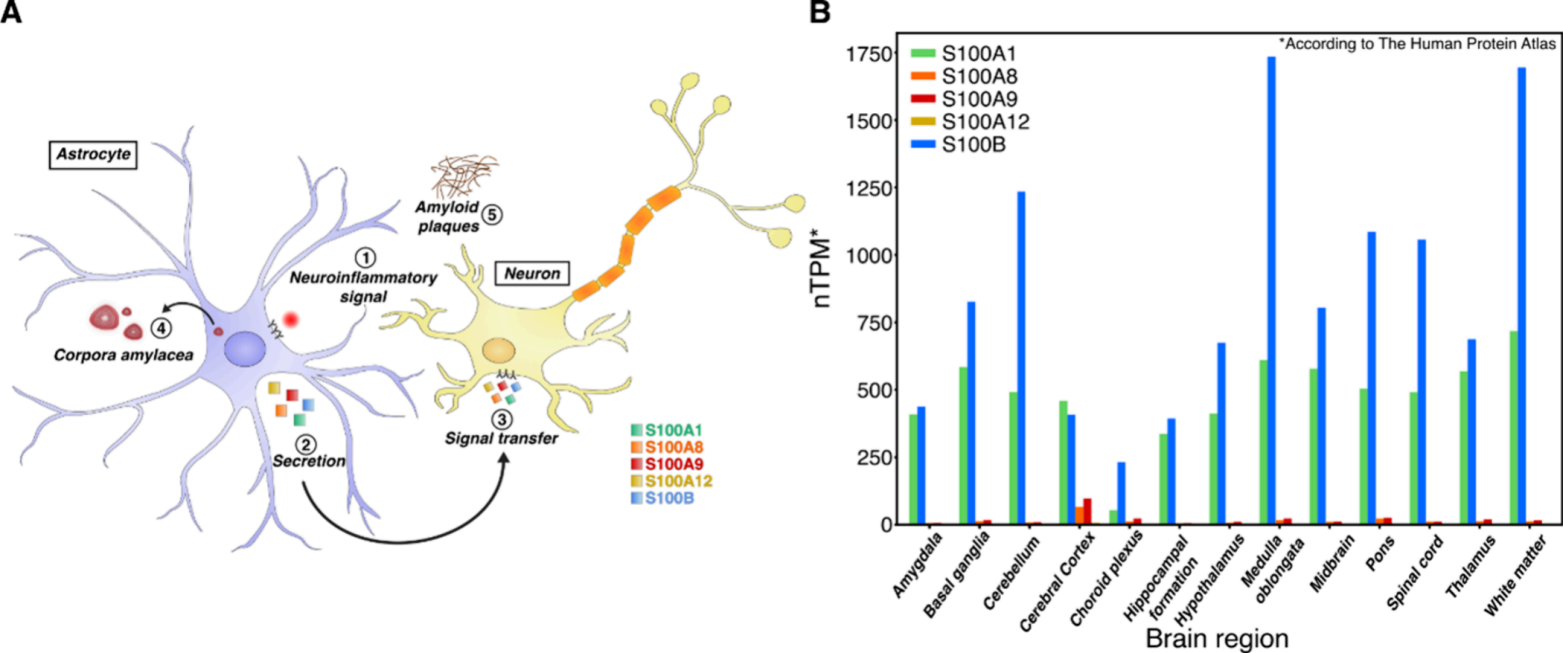
(A) During neuroinflammation (1), astrocytes upregulate the expression of S100A1, S100A8, and other S100 proteins.^7^ These proteins are secreted via noncanonical pathways^28^ (2) and transfer inflammation signals to glial or neuronal cells^2^ (3). S100 proteins can accumulate and contribute to the formation of corpora amylacea^11^ (4) and amyloid plaques^7^ (5). (B) Transcriptional differences of S100 RNA in various brain regions according to The Human Protein Atlas^15^ (nTPM, transcripts per million).

A hallmark feature of various neurodegenerative disorders is insoluble protein aggregates: amyloid fibrils^9^ and a number of S100 proteins form fibrils with distinct worm-like morphologies.^10^ Several S100 members can also accumulate in corpora amylacea (CA), glycoproteinaceous structures that appear in the aging brain and especially during neuropathologies (Figure 1A). The most abundant S100 proteins in CA are S100A1, S100A8, and S100A9,^11^ with S100A8 and S100A9 being shown to form amyloid fibrils or oligomeric complexes that are sensitive to amyloid dyes.^12^ Furthermore, S100A8 can form a heterodimer with S100A9 and assemble into calprotectin (CP), which stabilizes both proteins and prevents their aggregation.^12^ However, prolonged incubation of CP with zinc/calcium ions induces fibrillation.^13^ S100A1 protein is highly prevalent in the heart;^14^ however, it is also located in the brain, rivaling transcriptional levels to those of S100B, according to The Human Protein Atlas^15^ (Figure 1B), whereas S100A8 and S100A9 expression rises during neuroinflammation.^16^ S100B expression is known to correlate with neurodegenerative disorders;^17^ additionally, S100B can form a heterodimer with S100A1,^18^ but there is little information about S100A1’s role in neuropathologies. S100A1 is expressed in astrocytes^19^ (Figure 1A), can be found extracellularly,^20^ and is involved in neuroinflammation as a calcium ion sensor in the Alzheimer’s disease mouse model.^21^ Similarly to the S100B and S100A1 protein pair, there is a lot of information about S100A9’s role in neurodegeneration;^22,23^ however, there is a substantial lack of studies regarding its interaction partner S100A8. S100A8 is also produced in astrocytes (Figure 1A) and is linked to the enhanced production of beta-amyloid peptide through a positive feedback loop.^24^ Recently, we have shown that S100A8 formed various types of aggregates but not fibrillar structures, unlike several other S100 proteins.^10^ In general, only a few other S100 heterodimers are known,^18,25–27^ and their possible functions *in vivo* are not clearly understood. In this study, we investigated the interplay between S100A1 and S100A8 proteins and its impact on their stability as well as amyloid aggregation.

Our results revealed that S100A1 does not aggregate, and S100A8 forms only heterogeneous aggregates. Upon mixing the two proteins, we observed calcium concentration-dependent aggregation, resulting in fibril formation. At intermediate (50–400 μM) calcium concentrations, we observed worm-like fibrils, while higher (800–1600 μM) calcium concentrations inhibited aggregation. Fluorescence microscopy confirmed that both S100A1 and S100A8 colocalized to larger aggregate ensembles. However, double electron–electron resonance spectroscopy indicated no evident formation of a heterodimer, suggesting that the interactions are transient but substantial enough to affect the aggregation pathways. Overall, we confirmed that the mixture of S100A1 and S100A8 proteins leads to the formation of amyloid fibrils.

## Methods

### Cloning

The SUMO-S100A1 gene was purchased from GENEWIZ (Azenta Life Sciences). The gene was inserted into a pET28a(−) vector via the NdeI and BamHI restriction sites by standard cloning techniques,^29^ yielding a SUMO-S100A1 construct fused to an amino-terminal His6 tag. Primers for mCherry and eGFP genes were generated by a restriction free (RF) cloning tool (https://www.rf-cloning.org),^30^ and an RF cloning method^31^ was used to construct mCherry-S100A1 (pVK1) and eGFP-S100A8 (pVK2) plasmids in the pET28a(−) backbone vector. The plasmids and primers used in this study can be found in Table S1.

### Protein Expression and Purification

Plasmid constructs encoding 6xHis-SUMO-S100A1, 6xHis-SUMO-S100A8, 6xHis-mCherry-S100A1, and 6xHis-eGFP-S100A8 were transformed into One Shot BL21 Star (DE3) *Escherichia coli* (Thermo Scientific) cells by heat shock (42 °C for 45 s). Transformed cells were grown in 100 mL of LB medium containing kanamycin (50 μg/mL) at 37 °C and 220 rpm for 16 h. The culture was transferred to 200 mL of LB medium with kanamycin (50 μg/mL) and grown at 37 °C and 220 rpm until the optical density at 600 nm reached 0.6–0.8. Protein expression was induced by adding 200 μM IPTG, and the culture was incubated at 25 °C and 220 rpm for 18 h. Cells were harvested by centrifugation (6000*g*, 20 min, 4 °C). The biomass was resuspended in buffer (25 mM HEPES, 1.0 M NaCl, 10 mM imidazole, pH 8.0), which was followed by the addition of lysozyme and 1 mM phenyl­methyl­sulfonyl fluoride (PMSF). Cells were lysed by sonication (Sonopuls, VS70T probe; Bandelin) for 30 min at 40% amplitude, with a 15 s on/30 s off cycle. The lysate was centrifuged (18 000 rpm, 30 min, 4 °C), and the supernatant was filtered through a 0.45 μm pore size filter.

Protein purification via immobilized metal ion affinity chromatography (IMAC) was performed by using a gravity column packed with Ni^2+^ Sepharose 6 Fast Flow resin (Cytiva). The column was washed with 50 mM HEPES, 1.0 M NaCl, and 10 mM imidazole (pH 8.0) buffer, followed by elution using the same buffer solution that contained imidazole concentrations of 0.05 and 0.5 M imidazole. For all samples, the last fraction was collected.

6xHis-SUMO-S100A1 and 6xHis-SUMO-S100A8 were dialyzed (8000 Da MWCO, Biodesign D106) in 10 mM Tris buffer (pH 8.0) for 1 h. Sentrin-specific protease 1 (SENP1) was added to cleave the 6xHis-SUMO tag, and the samples were dialyzed for 18 h in fresh 10 mM Tris buffer (pH 8.0). Then, the samples were centrifuged (3160*g*, 30 min, 4 °C) and filtered through a 0.45 μm pore size filter. Purification via IMAC was repeated to collect the flow-through. All fractions were checked by SDS-PAGE. Before size exclusion chromatography, 10 mM EDTA and dithiothreitol (DTT) were added to all samples (S100A1, S100A8, 6xHis-eGFP-S100A8, and 6xHis-mCherry-S100A1). The samples were then concentrated (10 kDa MWCO, Merck) and filtered through a 0.22 μm pore size filter. Size exclusion chromatography was performed with columns packed with Superdex 75 sorbent (Cytiva) for S100A1 (10 546 kDa) and S100A8 (10 835 kDa) or Superdex 200 sorbent (Cytiva) for 6xHis-eGFP-S100A8 (40 935 kDa) and 6xHis-mCherry-S100A1 (40 227 kDa) proteins, calibrated with 50 mM HEPES (pH 7.4) buffer. The collected fractions were checked by SDS-PAGE and concentrated (10 kDa MWCO, Merck). The protein samples were stored at −80 °C.

### Differential Scanning Fluorimetry (DSF)

Samples for the protein stability assay contained either 100 μM S100A1, S100A8, or both S100A1/A8 prepared in 1 mM TCEP, 50 mM HEPES (pH 7.4) buffer with increasing CaCl_2_ concentrations (0, 50, 100, 200, 400, 800, 1600 μM) supplemented with 100 μM 8-anilino­naph­thal­ene-1-sulfonic acid (ANS). The ANS concentration was determined using its extinction coefficient (ε_351 nm_ = 5500 M^–1^ cm^–1^). For the sample without CaCl_2_, 1 mM EDTA was added. Additionally, S100A1 samples were prepared containing 0.5 and 1.0 M guanidinium chloride, without CaCl_2_. Protein unfolding was monitored with a Rotor-Gene Q instrument (QIAGEN) using the blue channel (excitation, 365 ± 20 nm; detection, 460 ± 20 nm). The unfolding process was initiated by ramping the temperature from 25 to 99 °C at 1 °C/min increments. Data analysis was performed using MoltenProt software.^32^

### Nano Differential Scanning Fluorimetry (nanoDSF)

S100A1 protein was desalted using a desalting column (BioWorks) with a 50 mM Tris buffer (pH 7.5). Prometheus NT.48 Series nanoDSF grade standard capillaries (NanoTemper Technologies) were filled with a sample containing 100 μM S100A1. Protein unfolding was monitored using a Prometheus NT.48 instrument (NanoTemper Technologies) by measuring the absorbance at 330 and 350 nm (with 20% excitation). The temperature was increased from 20 to 99 °C at a rate of 1 °C/min. Data analysis was performed using MoltenProt software.^32^

### Thioflavin T (ThT) Fluorescence Assay

The amyloid-specific Thioflavin T (ThT) dye was used to monitor the aggregation kinetics. Samples for the ThT fluorescence assay contained 1 mM TCEP, 50 μM ThT, and either 100 μM S100A1, S100A8, or S100A1/A8 in 50 mM HEPES buffer (pH 7.4). Each sample was prepared with increasing concentrations of CaCl_2_ (0, 50, 100, 200, 400, 800, 1600 μM). For the sample containing no CaCl_2_, 1 mM EDTA was added. 100 μL of each sample was placed into three separate wells of a 96-well nonbinding plate. Aggregation kinetics were measured every 5 min at 42 °C using a CLARIOstar Plus microplate reader. ThT dye was excited at 440 nm, and the emission signal was recorded at 480 nm.

### Densitometric Analysis

Samples after aggregation were either loaded directly onto a SDS-PAGE gel or first diluted 1:4 with 50 mM HEPES buffer (pH 7.4) from an initial 100 μM protein solution. The diluted samples were subsequently filtered through a 0.22 μm pore size filter before loading. Densitometric analysis of the SDS-PAGE gel was done using Image Lab software (Bio-Rad). The same diluted samples were also analyzed by Native-PAGE. Uncropped gel images are provided in the Supporting Information (Figure S1).

### Fourier Transform Infrared (FTIR) Spectroscopy

After 70 h of aggregation at 42 °C, samples were removed from the aggregation reaction kinetics plate (S100A8, 280 μL) or test tube (S100A1/A8, 1 mL) and were used for the preparation of FTIR measurements The aggregated samples were centrifuged at 16 9006g for 30 min, after which the supernatant was removed and replaced with 300 μL of D_2_O supplemented with 400 mM NaCl (the addition of NaCl may improve fibril sedimentation).^33^ The centrifugation and resuspension procedures were repeated four times. After the final step, the aggregate pellet was resuspended into 50 μL of D_2_O containing 500 mM NaCl.

FTIR spectra were acquired as described previously^34^ using an Invenio S FTIR spectrometer (Bruker), equipped with a liquid nitrogen-cooled mercury-cadmium-telluride detector, at room temperature with constant dry-air purging. For every sample, 256 interferograms with 2 cm^–1^ resolution were recorded and averaged. D_2_O containing 400 mM NaCl and water vapor spectra were subtracted from each sample spectrum, followed by baseline correction and normalization to the same 1595–1700 cm^–1^ wavenumber range. All data processing was performed using GRAMS software.

### Far-UV Circular Dichroism (CD) Spectroscopy

Samples for CD spectral measurements contained either 100 μM S100A1, S100A8, or S100A1/A8 in a 1 mM TCEP, 50 mM HEPES buffer (pH 7.4). Each sample was prepared with increasing concentrations of CaCl_2_ (0, 50, 100, 200, 400, 800, 1600 μM). For the sample without CaCl_2_, 1 mM EDTA was added. The aggregation of samples was conducted in 1.5 mL microcentrifuge tubes (Eppendorf) at 42 °C for 70 h. The samples were then placed in a 0.5 mm quartz cuvette, and CD spectra were measured using a J-815 spectropolarimeter (Jasco). For each sample, spectra between 190 and 260 nm were recorded at 0.2 nm intervals. The data were smoothed using Gaussian smoothing (SD = 10). All data processing was done using Quasar.^35^

### Atomic Force Microscopy (AFM)

The freshly cleaved mica was positively charged by applying 50 μL of 0.5% (3-amino­propyl)­tri­ethoxy­silane (APTES) on the surface and allowing it to functionalize for 5 min. Subsequently, mica was washed with dH_2_O and dried with airflow, and the procedure was repeated with 50 μL of the protein sample, which was prepared by diluting it 1:9 with HEPES (pH 7.4) buffer. Imaging was performed using a Dimension Icon microscope (Bruker) operating in tapping-in-air mode with aluminum-coated silicon tips (RTESPA-300, Bruker). The images were processed using Gwyddion 2.66 software.^36^ The cross-sectional height of aggregates was determined from extracted line profiles, which were fitted by using the Gaussian function. Examples of cross-sectional height profiles are presented in the Supporting Information (Figure S2).

### Fluorescence Microscopy

The samples for fluorescence microscopy were prepared as in the ThT fluorescence assay with the exclusion of ThT dye and the addition of either or both 1 μM mCherry-S100A1 and eGFP-S100A8 proteins, resulting in 99 μM nontagged and 1 μM tagged proteins, respectively.

15 μL aliquots of each sample were pipetted onto 1 mm glass slides (Fisher Scientific, cat. no. 11572203), covered with 0.18 mm coverslips (Fisher Scientific, cat. no. 17244914), and imaged as described previously^37^ using an Olympus IX83 microscope with a 40× objective (EVIDENT, NA 0.6, LUCPLFLN40X) and fluorescence filter cubes (475–495 nm excitation and 510–550 nm emission wavelengths for eGFP-S100A8; 540–550 nm excitation and 575–625 nm emission wavelengths for mCherry-S100A1). Images were captured using an ORCA-Fusion Digital CMOS camera (Hamamatsu, model C14440-20UP). Data analysis was done using Fiji software.^38^ Non-cropped fluorescence images are presented in Figures S3–S5.

### Transmission Electron Microscopy (TEM)

On glow-discharged 300-mesh Formvar/carbon supported copper grids (Agar Scientific), 5 μL of each sample prepared for fluorescence microscopy was applied and incubated for 1 min; then, the grid was dried with filter paper. The same procedure was repeated with 5 μL of 2% (w/v) uranyl acetate, followed by two washes with 5 μL of dH_2_O. TEM images were acquired using a Talos 120C (Thermo Fisher) microscope operating at 120 kV, equipped with a 4k × 4k Ceta CMOS camera. Images were processed using Fiji software.^38^

### Chaperone Activity Assay

Samples were prepared by mixing 0.2 mg/mL lysozyme with either 10 μM S100A1, 10 μM S100A8, or both 10 μM S100A1/A8 in 50 mM HEPES (pH 7.4) buffer containing 20 mM DTT. Three samples of each mixture were prepared with increasing concentrations of CaCl_2_ (0, 20, 200 μM). For the samples that did not contain CaCl_2_, 2 mM EDTA was added as a control to ensure bivalent metal chelation. 100 μL of each sample was placed into four separate wells of a 96-well nonbinding plate. The chaperone activity of S100A1, S100A8, and S100A1/A8 was measured spectrophotometrically by monitoring the absorbance of lysozyme aggregates at 360 nm using a CLARIOstar Plus microplate reader at 37 °C.^39^

### AlphaFold Prediction

Protein structures were predicted using AlphaFold3.^40^ The amino sequences of S100A8 and S100A1 with or without 4 atoms of calcium ions were used as inputs and run with default settings. S100A8/S100A1 confidence scores were ipTM = 0.8 and pTM = 0.81 in the presence of calcium ions. Confidence levels and scores are depicted in Figure S6.

### Electron Paramagnetic Resonance (EPR)

For electron paramagnetic resonance (EPR) double electron–electron resonance (DEER) measurements, proteins were labeled as described previously.^41^ Briefly, S100A1 or S100A9 were incubated with 10 mM DTT for 1 h and desalted using a BabyBio desalting column (Bio-Works) with PBS. Next, proteins were incubated with 10 times excess of nitroxide spin label MTSSL (Sigma-Aldrich) reagent overnight at 8 °C with gentle shaking. Proteins were desalted again to 50 mM HEPES 10% glycerol (pH 7.4) buffer in the morning. The final concentrations of labeled S100A1 and S100A9 proteins were 50 and 100 μM, respectively. For heterodimer measurements, proteins were mixed equimolarly with their partners.

DEER spectroscopy measurements were performed at the X-band (9.5 GHz) microwave frequency by using a Bruker ELEXSYS E580 EPR spectrometer. The sample was cooled to 50 K using liquid helium in a helium flow cryostat. A 4 mm diameter sample tube containing approximately 20 μL of sample was flash-frozen in liquid nitrogen and subsequently inserted into a Bruker ER4118X-MD5 microwave resonator. For improved sensitivity, a cryoprobe equipped with a cryogenic low-noise microwave amplifier was employed.^42^

An echo-detected field-sweep EPR spectrum was recorded by using a Hahn-echo pulse sequence. The pulse sequence for the four-pulse DEER experiment was

$$\frac{\pi_{\mathrm{obs}}}{2}-\tau_{1}-\pi_{\mathrm{obs}}-t_{1}-\pi_{\mathrm{pump}}-\left(\tau_{1}+\tau_{2}-t_{1}\right)-\pi_{\mathrm{obs}}-\tau_{2}-\mathrm{echo}$$

Measurements were acquired with an interpulse delay τ_1_ of 400 ns for S100A1 and 300 ns for S100A9 samples. The dead time delay *t*_1_ of 80 ns was used for all samples, while the interpulse delay τ_2_ and the shot repetition time were optimized for each sample. The microwave power was optimized to obtain a π/2-pulse length of 16 ns and a π-pulse length of 32 ns for both pump and observer pulses. All traces were collected using eight-step nuclear modulation averaging, with an averaging time step of 56 ns, a 20 ns time-domain for the primary data, and a 60 MHz frequency difference between the observer and pump pulses. The data analysis was performed using DeerLab^43^ (release 1.1.2), a Python-based package for DEER spectroscopy data analysis. The experimental signal was modeled using the general kernel, assuming a homogeneous spin distribution in a 3D medium for the background. The distance distribution was fitted using a Tikhonov regularization with the regularization penalty weight selected using the Akaike information criterion and a non-negativity constraint. The asymptotic method was used to determine the 95% confidence intervals. The DeerLab results were also compared to other software tools, including DeerAnalysis 2022 ^44^ and DeerNet,^45^ all of which produced highly consistent outcomes.

## Results and Discussion

### S100A1 Alters Aggregation of S100A8

The aggregation of S100A1 and S100A8 was followed by the amyloid-specific fluorescent ThT dye.^46^ Aggregation was not observed for S100A1 with or without calcium ions (Figure 2A), whereas S100A8 aggregated in a two-phase manner and calcium extended the duration of the first phase (Figure S7), consistent with previous findings.^12^ When S100A1 and S100A8 were mixed in equimolar concentrations, the aggregation kinetics changed, depending on calcium concentration. At CaCl_2_ concentrations up to 50 μM, proteins aggregated in a biphasic manner, but at higher calcium ion concentrations, the aggregation transitioned to an exponential growth phase, resembling the kinetics observed for S100A9.^47^ Comparing the maximum ThT fluorescence levels at the end of the aggregation process (Figure 2B), S100A8 aggregation fluorescence exponentially decreased with increasing calcium ion concentration, but the intensities of the S100A1/S100A8 samples were higher, presumably due to calcium ion binding competition between S100A1 and S100A8 proteins. At the highest CaCl_2_ concentration of 1600 μM, aggregation was strongly inhibited, hinting that both calcium-binding EF-hands of S100A1 and S100A8 were saturated with calcium ions, leading to stabilized native structures. This was further confirmed with the densitometry analysis of aggregated samples by SDS-PAGE (Figure 2C). We also observed that at lower CaCl_2_ concentrations, most of S100A8 aggregated, whereas only one-third of S100A1 aggregated. Native-PAGE of the same samples indicated no formation of heterodimers, but smear bands observed for the S100A8 protein indicate heterogeneous mixtures, likely resulting from conformational changes, oligomerization, or aggregation (Figure S1C).

**Figure 2:**
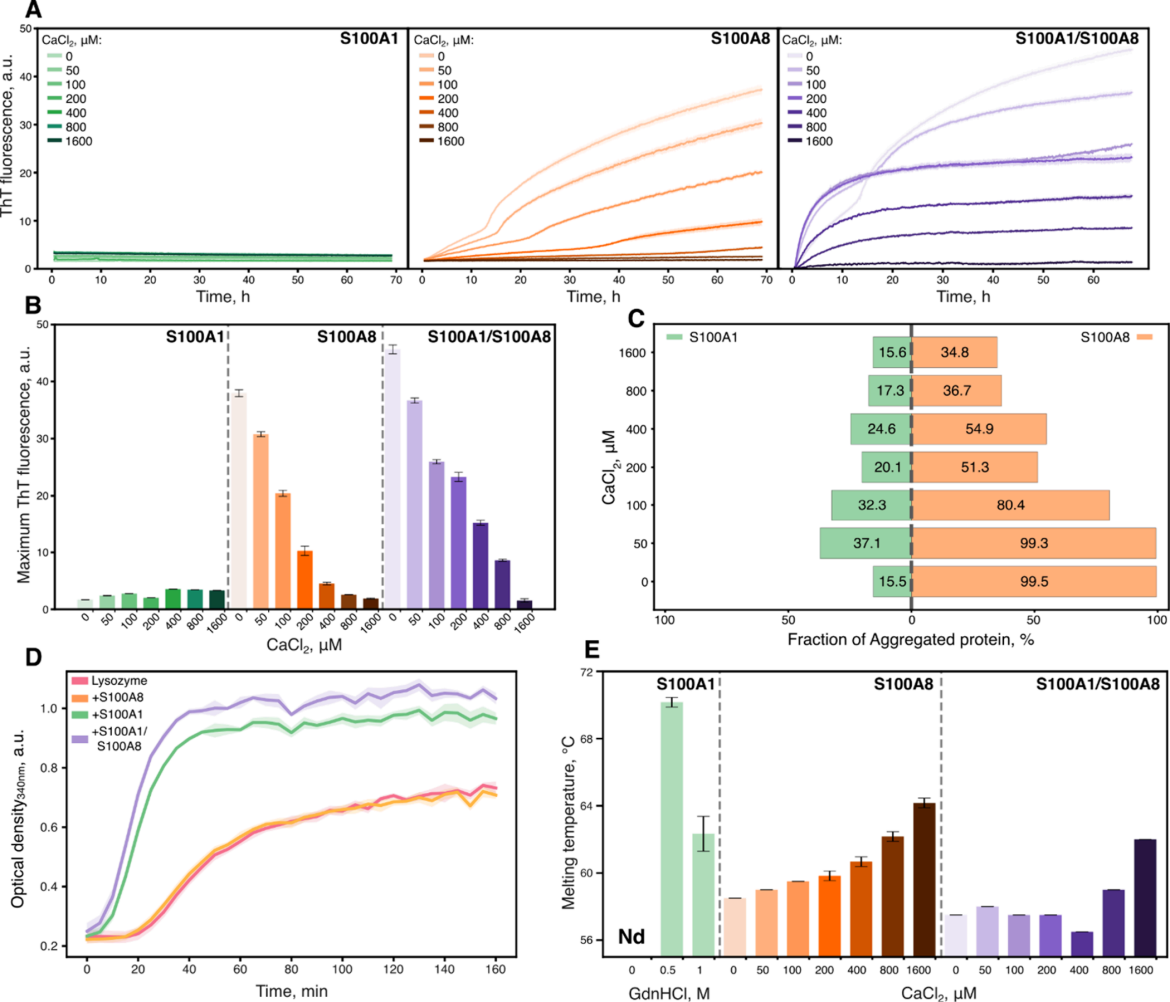
Aggregation and stability propensities of S100A1 and S100A8 proteins. (A) Aggregation kinetics of S100A1, S100A8, and S100A1/S100A8 in the presence or absence of calcium ions, followed by ThT fluorescence. (B) Maximum ThT fluorescence values of aggregation kinetics. (C) Densitometric quantification of filtered S100A1, S100A8, and S100A1/S100A8 samples after aggregation. (D) Chaperone assay of S100A1 (10 μM), S100A8 (10 μM), and S100A1/S100A8 (10 μM each) against DTT-induced aggregation of lysozyme (0.2 mg/mL). (E) Melting temperatures of S100A1, S100A8, and S100A1/S100A8 proteins.

We also investigated whether S100A1 exhibits any chaperone activity that could influence aggregation since it is known to form a multichaperone complex with Hsp70/90,^48^ and other S100 members have also been reported to have chaperone-like functions.^49,50^ In the lysozyme chaperone activity assay (Figure 2D), S100A1 or a mixture of both proteins accelerated the aggregation of lysozyme, consistent with previous reports for S100A6,^51^ whereas S100A8 had no effect. Notably, the presence of calcium ions inhibited the aggregation of lysozyme (Figure S8), but in the mixture with S100A1 or S100A1/S100A8, aggregation was observed, due to calcium ions being salvaged by S100A proteins. In higher calcium concentrations (Figure S8), lysozyme aggregation was inhibited under all conditions, preventing further analysis of chaperone-like activity. These findings suggest that, unlike the expected chaperone activity, S100A1 interactions may promote coaggregation or modify aggregation pathways through interactions with other proteins.

Concurrently, we investigated the protein melting temperature using differential scanning fluorimetry by measuring the fluorescence of the ANS dye (Figure S9), which binds to hydrophobic pockets upon protein unfolding or aggregation.^41^ We did not observe the unfolding of S100A1 under initial conditions; only upon the addition of a denaturing reagent (guanidine hydrochloride) were noticeable melting temperatures obtained (Figure 2E). This contrasts with previously reported S100A1 melting temperatures, which ranged from 70 to 75 °C when measured in Tris buffer without guanidine hydrochloride using nanoDSF.^52^ Therefore, we repeated the measurement under those conditions (Figure S10) and observed S100A1 melting at a slightly higher temperature of 85 °C, which might be due to different protein preparations. Since nanoDSF measures protein changes via aromatic residue shifts,^53^ there is a possibility that it detects intrinsic conformational changes, partial unfolding, or monomerization, which DSF does not detect. However, we did observe denaturation of S100A8 using DSF, and calcium ions stabilized S100A8 as reported previously.^12,54^ In the mixture of both proteins, melting temperatures were slightly lower and increased at higher calcium ion concentrations (>400 μM), suggesting potential competition for calcium binding or transient interactions between the proteins.

### S100A1/S100A8 Amyloid Fibrillation Is Dependent on Calcium Concentration

We investigated the aggregates formed by S100A1 and S100A8 by using three different microscopy techniques. To begin with, atomic force microscopy revealed the distinct morphologies of assemblies. S100A1 formed a small number of amorphous aggregates, whereas S100A8 aggregated into spherical oligomers/clusters (Figure 3A). Upon the addition of calcium ions, fewer aggregates were observed in the S100A8 sample, correlating with the lower fluorescence of ThT in the aggregation kinetic data (Figure 2A) and previously published results.^12^ Although in the absence of calcium ions the S100A1/S100A8 mixture resembled S100A8 aggregates, worm-like fibrils started to form upon the addition of calcium ions (Figure 3B). The size of the aggregates steadily increased from 2.14 to 2.86 nm with calcium ion concentrations up to 400 μM (Figure 3C). Worm-like fibrils indicate that S100 proteins potentially only partially unfold, leading to curly fibrils, similar to lysozyme fibrils.^55^ To support observations by AFM, additional images with a transmission electron microscope (Figure 3D) were acquired using nondiluted samples, unlike those prepared for AFM. Samples of S100A1 and S100A8 either contained no aggregates or formed amorphous assemblies, respectively (Figure S11A). At 200 μM CaCl_2_, worm-like fibrils were observed, in correspondence with the AFM results. Surprisingly, at the highest CaCl_2_ concentration (1600 μM), fibrils were also detected, although they seemed more likely to cluster. This was also confirmed with AFM, using the same protein concentration (100 μM) for imaging (Figure S11B).

**Figure 3:**
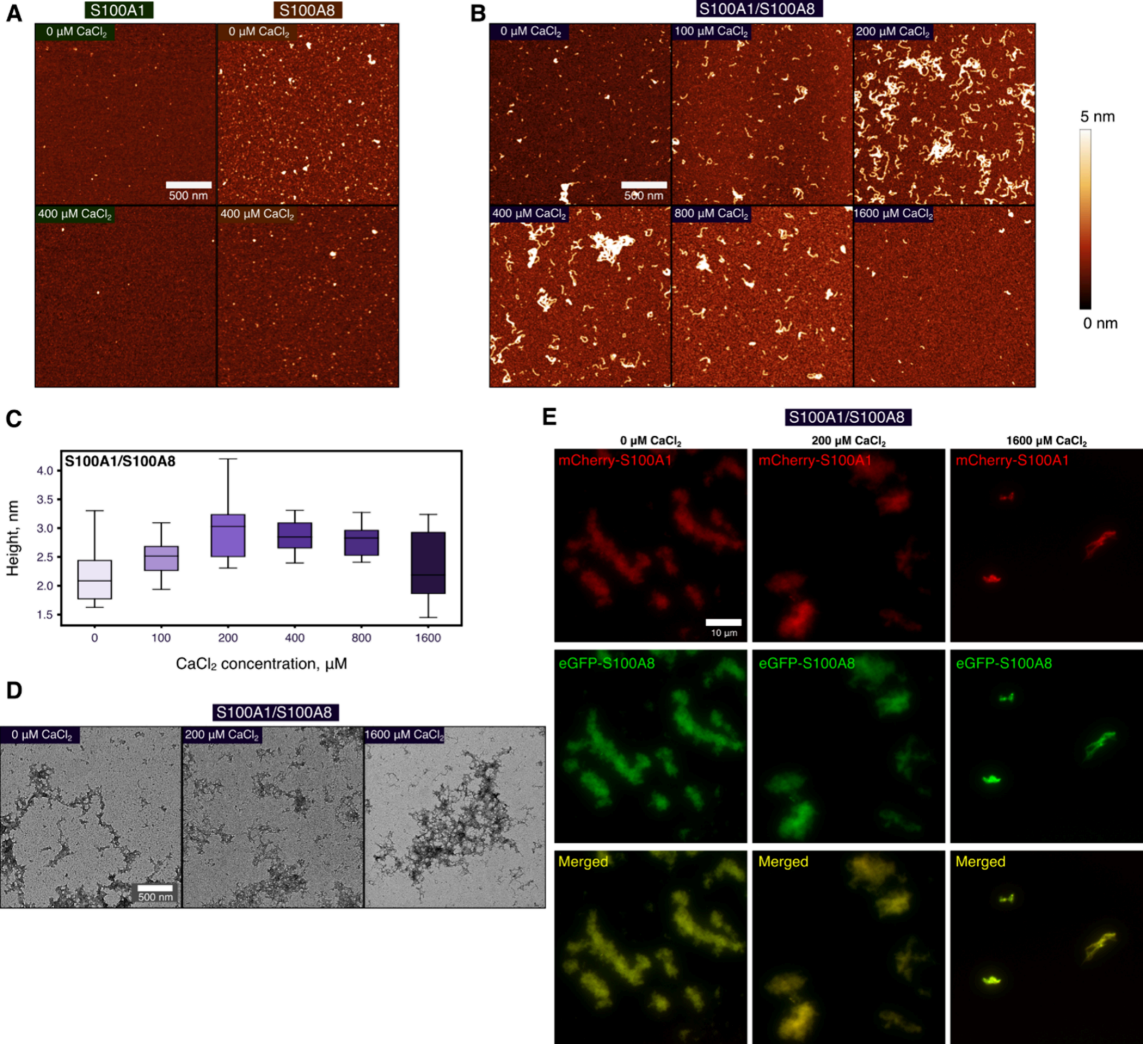
Morphology and colocalization of S100A1 and S100A8 aggregates. AFM images of (A) S100A1, S100A8, and (B) S100A1/S100A8 after 70 h of aggregation (scale bar: 500 nm). (C) The height distribution of S100A1/S100A8 samples with box plots indicating the median, interquartile range (IQR), and whiskers 1.5× range of IQR from box. (D) Transmission electron microscopy (scale bar: 500 nm) and (E) fluorescence microscopy (scale bar: 10 μm) images of S100A1/S100A8 aggregated mixtures at different calcium ion concentrations. Additional fluorescence images are presented in Figures S3–S5.

Finally, fluorescence microscopy was employed to investigate whether S100A1 and S100A8 colocalize (Figure 3E, Figure S11C). At all ranges of calcium concentrations, we observed colocalization of both proteins in large plaques. The largest difference was at 1600 μM CaCl_2_, where only much smaller and fewer clumps were seen, which is expected due to aggregation inhibition. Altogether, in the mixture of both proteins, worm-like fibrils are formed and both proteins can colocalize, even at the highest calcium concentration.

### Structural Properties of S100A1/S100A8 Complex

After imaging, we conducted structural investigations of S100A1 and S100A8 proteins. First, we examined their structure postaggregation using circular dichroism, which takes into account all conformations of proteins in solution. S100A1 exhibited globular conformation in all conditions with two minimums at 209 and 222 nm (Figure 4A), indicating an α-helical structure.^56^ S100A8, in the absence of calcium ions, displayed aggregation into β-sheets. Aggregates were separated from soluble monomers, and their secondary structure was further confirmed by FTIR spectroscopy (Figure S12), showing an amide I band at ∼1620 cm^–1^,^57^ in correspondence with previously reported S100A8 and S100A9 aggregates.^12,47^ The addition of calcium ions steadily stabilized S100A8 into an α-helical fold. Although we observed reduced ellipticity for the mixture of both proteins in lower calcium ion concentration ranges, the spectra consistently resembled an α-helical structure throughout all tested conditions. Since only a smaller portion of protein aggregated according to densitometry results (Figure 2B), β-sheets of aggregates might be invisible due to the major fraction of soluble protein. Using a larger volume of initial sample, we were able to measure the FTIR spectra (Figure S12) of separated S100A1/S100A8 aggregates prepared with 200 μM CaCl_2_. It confirmed that they do form β-sheet structures, similar to aggregates formed in the absence of calcium ions.

**Figure 4:**
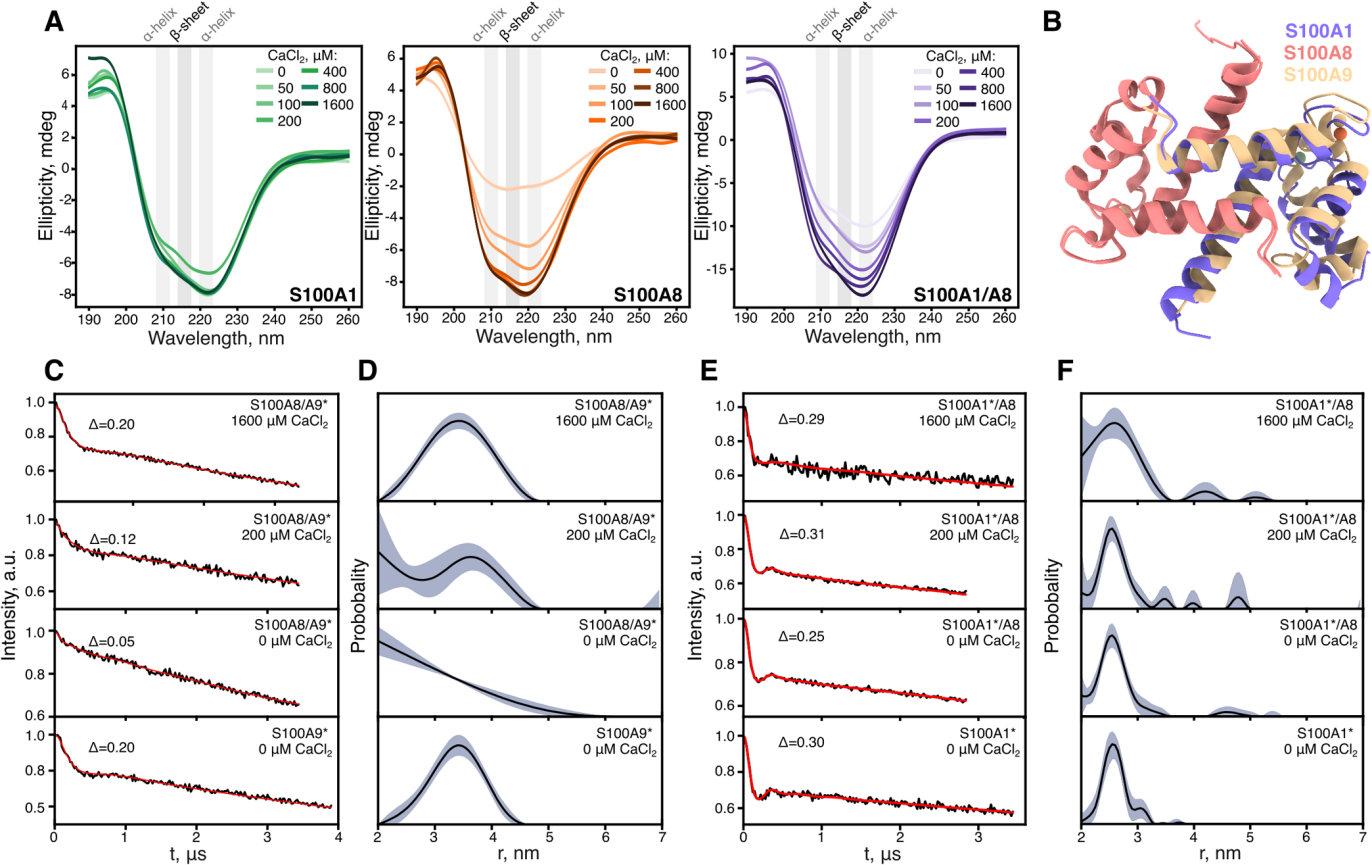
Structure and cross-interaction of S100A1 and S100A8 proteins. (A) CD spectra of S100A1, S100A8, and a mixture of both proteins after 70 h of aggregation at 42 °C. (B) Predicted AlphaFold model of S100A1 and S100A8 heterodimer compared to S100A8/S100A9 heterodimer (PDB ID: 1XK4). Time-domain DEER signals and regularized distance distributions of S100A8/S100A9 (C, D) and S100A1/S100A8 (E, F) samples at different calcium concentrations. MTSSL spin-labeled proteins are indicated by an asterisk (*).

To determine potential heterodimer formation between S100A1 and S100A8, we predicted their structure using AlphaFold3 Web server^40^ and compared them to the known S100A8/S100A9 heterodimer (Figure 4B). In both heterodimers, S100A8 maintained an identical conformation, while S100A1 adopted a structure similar to that of S100A9, with minor alterations in the loops. On the whole, the hypothetical heterodimer of S100A1/A8 resembled S100A8/S100A9.

To further investigate the potential formation of the S100A1/S100A8 heterodimer and compare it to characterized S100A8/S100A9, we used DEER spectroscopy. With DEER, we measured distances of spin-labeled cysteines in S100A9 and S100A1 proteins, which were mixed with nonlabeled S100A8. The cysteine of S100A9 is located at the N-terminus and S100A1 at the C-terminus, thus allowing accurate measurement of protein diameters (Figure S13). First, we observed 3.45 nm with a fwhm of 0.5 nm distance for the S100A9 homodimer and no signal for the S100A8/S100A9 complex (Figure 4C,D), which corresponds to the successful formation of the heterodimer. The signal was recovered with the addition of calcium, indicating dissociation of heterodimers. It is known that S100A8/S100A9 forms tetramers in the presence of calcium ions; however, it requires refolding of both proteins at the same time, as mixing them is not sufficient,^58^ a process we performed in this study. Overall, by employing DEER spectroscopy, we were able to detect the S100A8/S100A9 heterodimer; thus, we followed up experiments with S100A1 and S100A8. In all conditions (Figure 4E,F), we observed distances of 2.55 nm, which is close to the S100A1 homodimer diameter (Figure S13). Altogether, even though AlphaFold3 predicted the S100A1/S100A8 heterodimer, S100A1 and S100A8 do not form dimers, or their population is too low to be detected by DEER spectroscopy; therefore, the likely interaction during coaggregation is between different homodimers or in larger oligomeric states.

## Conclusions

The S100 family consists of small proteins with a basic structure, yet these proteins exhibit a plethora of functions^2^ and play a major role in activating neuroinflammation, as well as the progression of diseases.^8^ In correlation with their large expression in the brain, they have been identified to coaggregate or interact with neurodegenerative disorder related proteins, such as amyloid-beta,^59^ alpha-synuclein,^60^ or tau.^49^ Despite this, relatively little is known regarding the interactions among different family members, although they are known to oligomerize and form heterodimers.^54,61,62^ Furthermore, there is even less understanding of how transient or weak interactions can determine the protein structure or aggregation pathways.

In this study, we have shown that S100A1 affects S100A8 aggregation. Unlike S100A8 or S100A9, S100A1 is highly stable and aggregates minimally; however, it can interact with S100A8, which leads to coaggregation and the formation of worm-like fibrils. Calcium ions further mediate these interactions from promoting to inhibiting aggregation at the highest concentration. Inside the cell, calcium levels are around 100 nM,^63^ while extracellular concentrations are approximately 1 mM;^64^ therefore, coaggregation might occur when S100 proteins are released into the extracellular space. On the other hand, during calcium influx, the calcium concentration can increase up to 1 μM and higher ranges,^65^ enabling interactions between S100 proteins. These interactions are difficult to observe, but we have identified that they might be critical to protein stability and structural changes. In general, calcium showed a dual role: while it promotes the stability of S100 proteins, it can also lead to aggregation due to increased, albeit still weak, interactions between proteins.

Another conclusion of the results is that, similarly to moonlight proteins,^66^ which catalyze biochemical processes through weak protein or surface interactions, S100A1 allows the formation of S100A8 worm-like fibrils. While S100A1 is known to have cochaperone activity,^48^ which promotes interactions with aggregating proteins such as S100A8, it is insufficient to fully suppress aggregation. Instead, it inhibits the amorphous aggregation of S100A8 while promoting amyloid formation. S100A1 does not appear to protect against aggregation, leading to antichaperone activity and colocalization. Similarly, S100 proteins are found together in aggregate deposits in the brain,^11^ which might occur due to the same transient interactions. Moreover, since S100A1 is expressed (Figure 1B) constantly and S100A8 expression increases during neuroinflammation,^16^ the aggregation of proteins could only occur during neurodegenerative disease onset or progression. Our study provides a new perspective on S100A1 and S100A8 protein interactions and expands our understanding of them, which have yet to be sufficiently investigated compared to their other protein partners.

## Supplementary Material



## Data Availability

The raw data used in this paper have been tabulated and are available on Mendeley Data: 10.17632/rr75p7h9f7.1. All other relevant data are available from the corresponding author upon reasonable request.

## Notes

SDS-PAGE gels; additional fluorescence microscopy, AFM, and TEM images; thermal protein denaturation profiles; FTIR spectra; AlphaFold3-predicted structure confidence levels; chaperone assays in the presence of calcium; and primers used in this study (PDF)
