# Supplementary material for: Calcium-Dependent S100A8 Amyloid Fibril Formation via S100A1-Mediated Transient Interaction
Source: ACS Chem Neurosci. 2025 Jun 25;16(14):2592–601. doi: 10.1021/acschemneuro.5c00086 (PMC12272555; doi:10.1021/acschemneuro.5c00086)
Supplement: Supplementary file 1 [file cn5c00086_si_001.pdf]

Supporting information for

Calcium-Dependent S100A8 Amyloid Fibril Formation via S100A1-mediated Transient Interaction

Viktorija Karalkevičiūtė[1], Ieva Baronaitė[1], Aistė Peštenytė[2], Dominykas Veiveris[1], Gediminas Usevičius[2], Mantas Šimėnas[2], Mantas Žiaunys[1], Vytautas Smirnovas[1], Darius Šulskis[1*]

[1]Institute of Biotechnology, Life Sciences Center, Vilnius University, LT-10257 Vilnius, Lithuania

[2]Faculty of Physics, Vilnius University, LT-10222 Vilnius, Lithuania

*Correspondence should be addressed to

Email: darius.sulskis@gmc.vu.lt

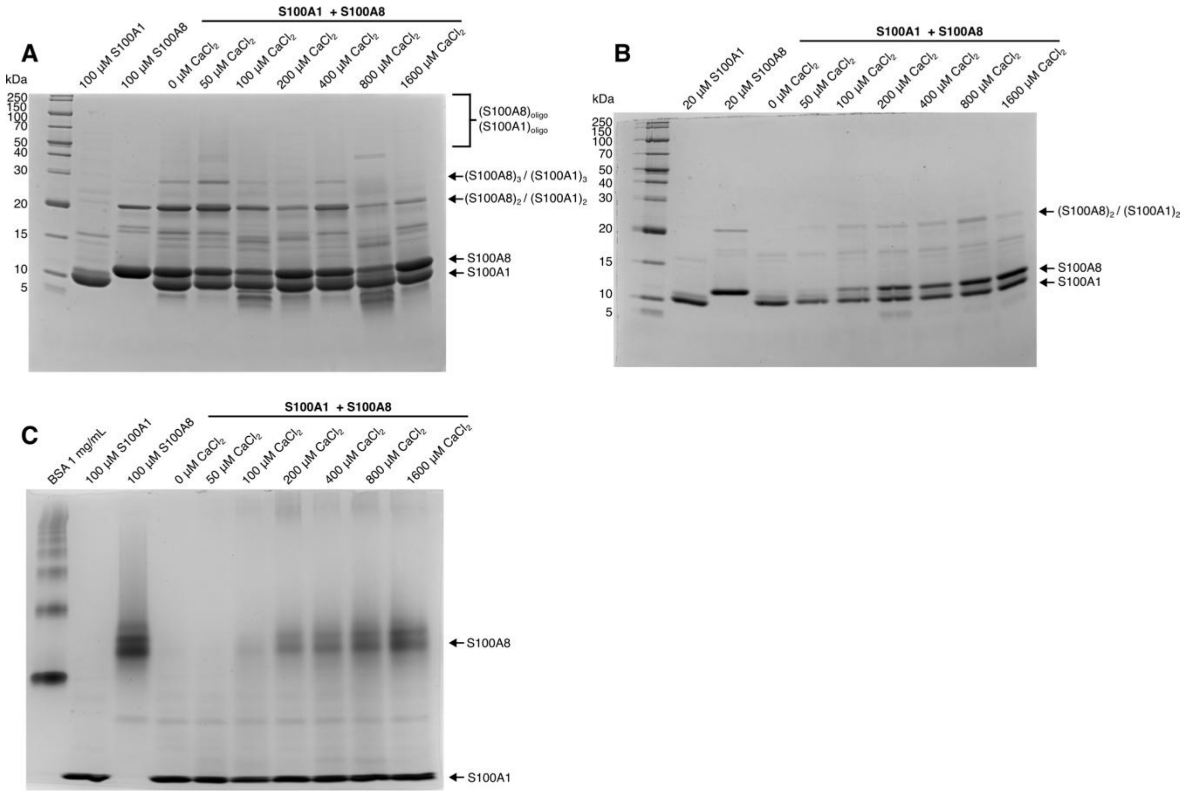

Figure S1. SDS-PAGE of unfiltered (**A**), SDS-PAGE (**B**) or native-PAGE (**C**) of filtered S100A1/S100A8 samples prepared under different $CaCl_2$ concentrations after 70 h of aggregation at 42 °C.

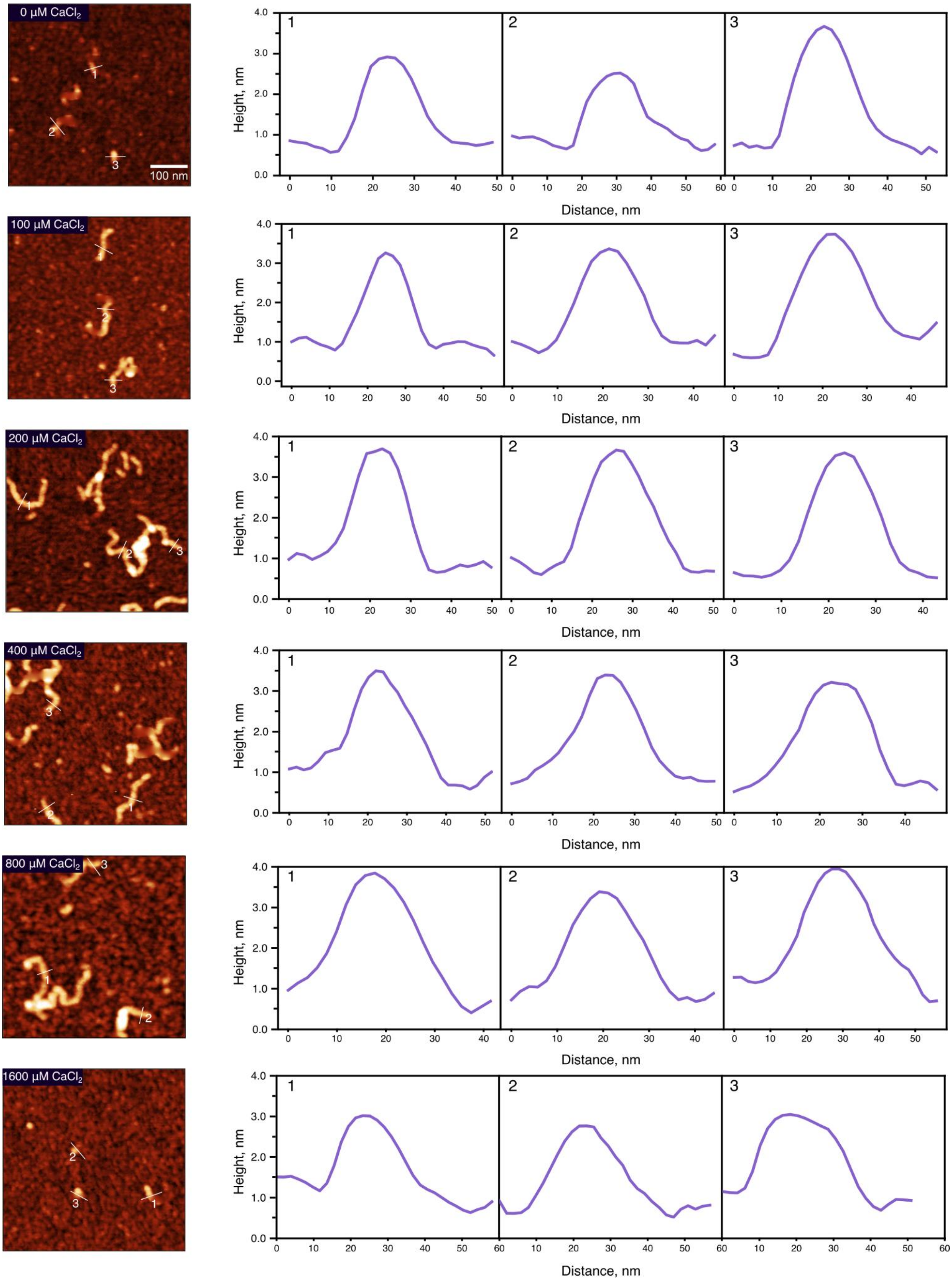

Figure S2. The representative height profiles of S100A1/A8 particles from AFM images (scale bar 100 nm).

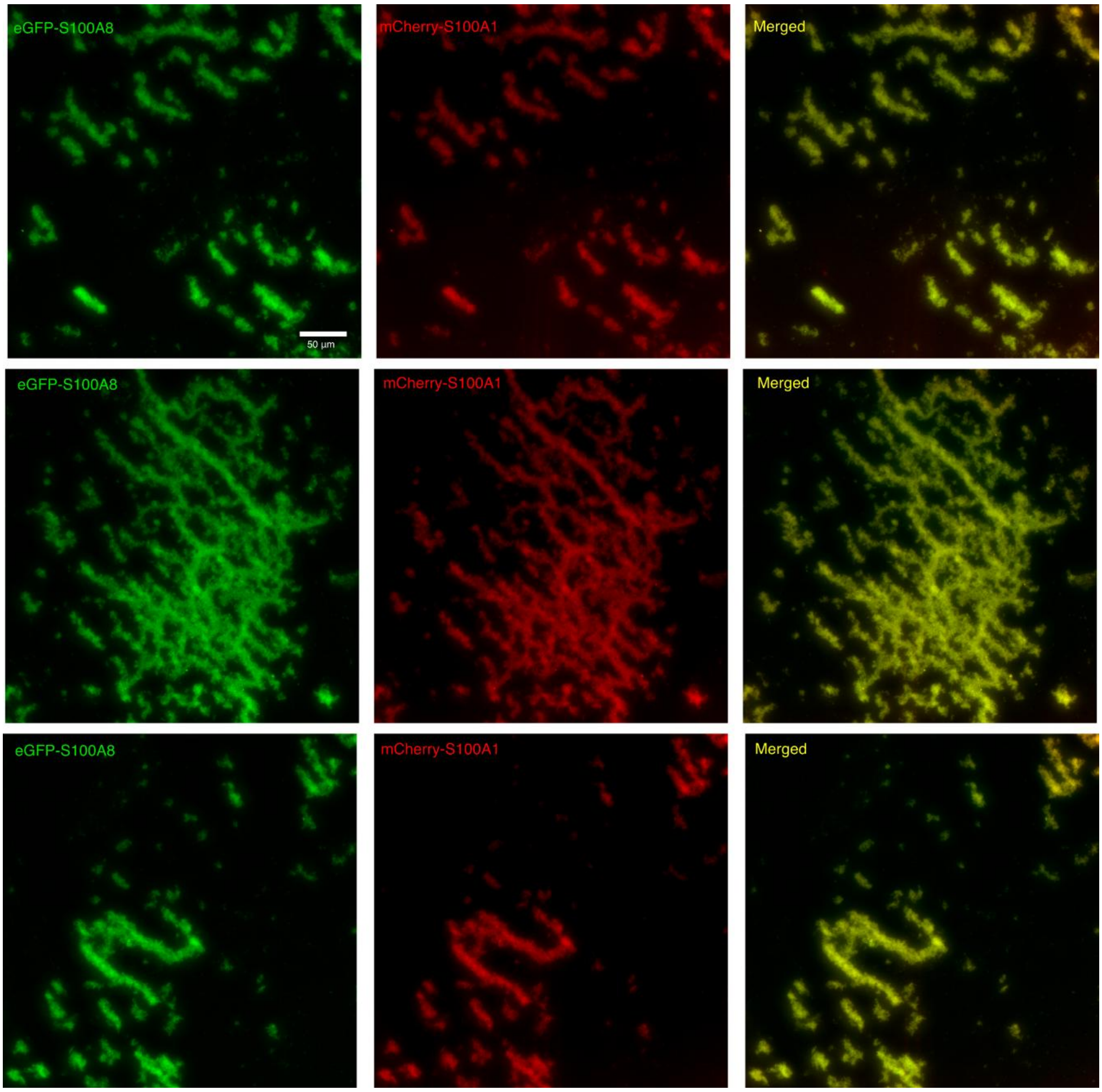

Figure S3. Fluorescence microscopy images of S100A1/S100A8 sample after aggregation without $CaCl_2$. Scale bar is 50 μm.

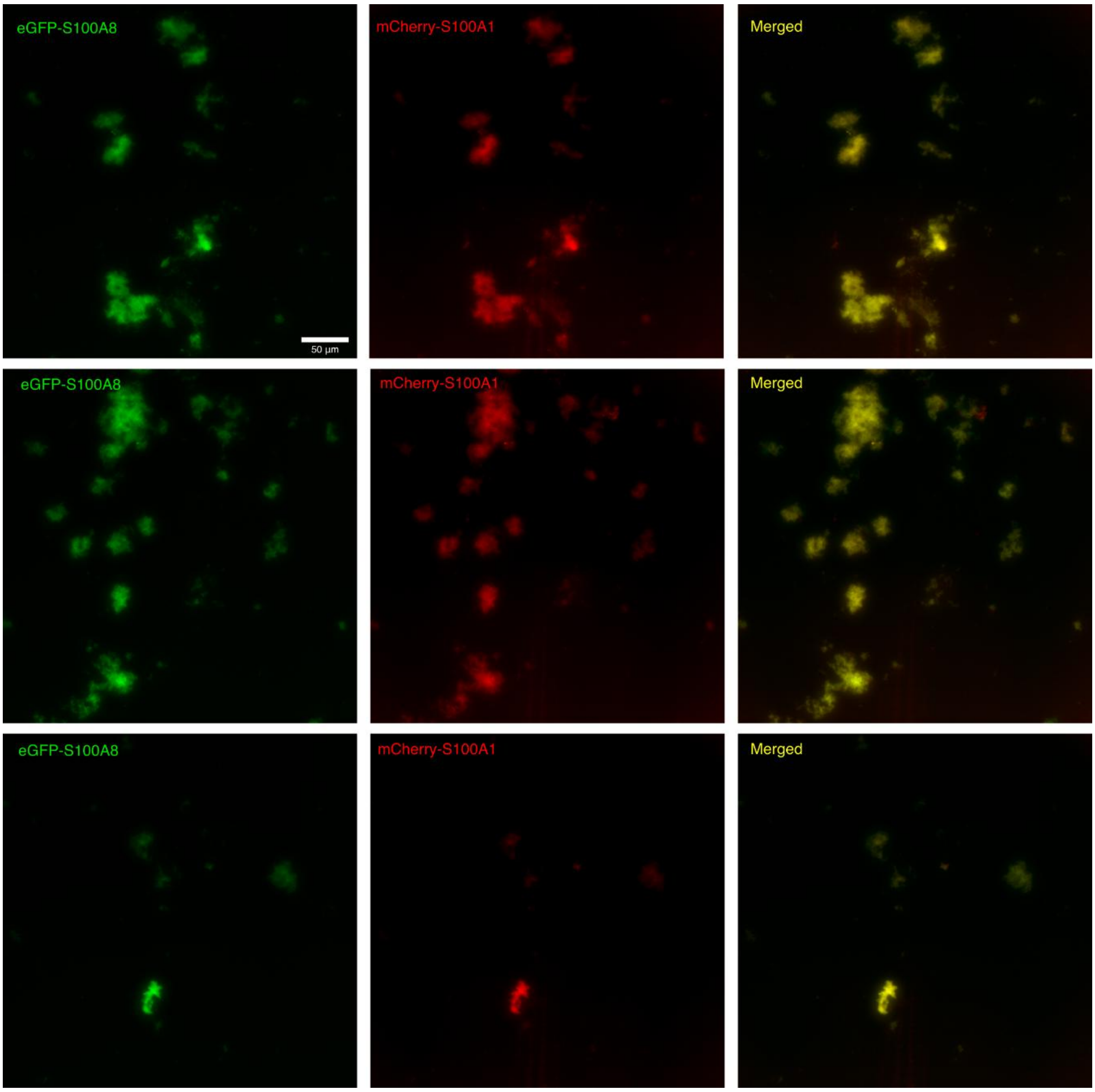

Figure S4. Fluorescence microscopy images of S100A1/S100A8 sample after aggregation in the presence of 200 μM $CaCl_2$. Scale bar is 50 μm.

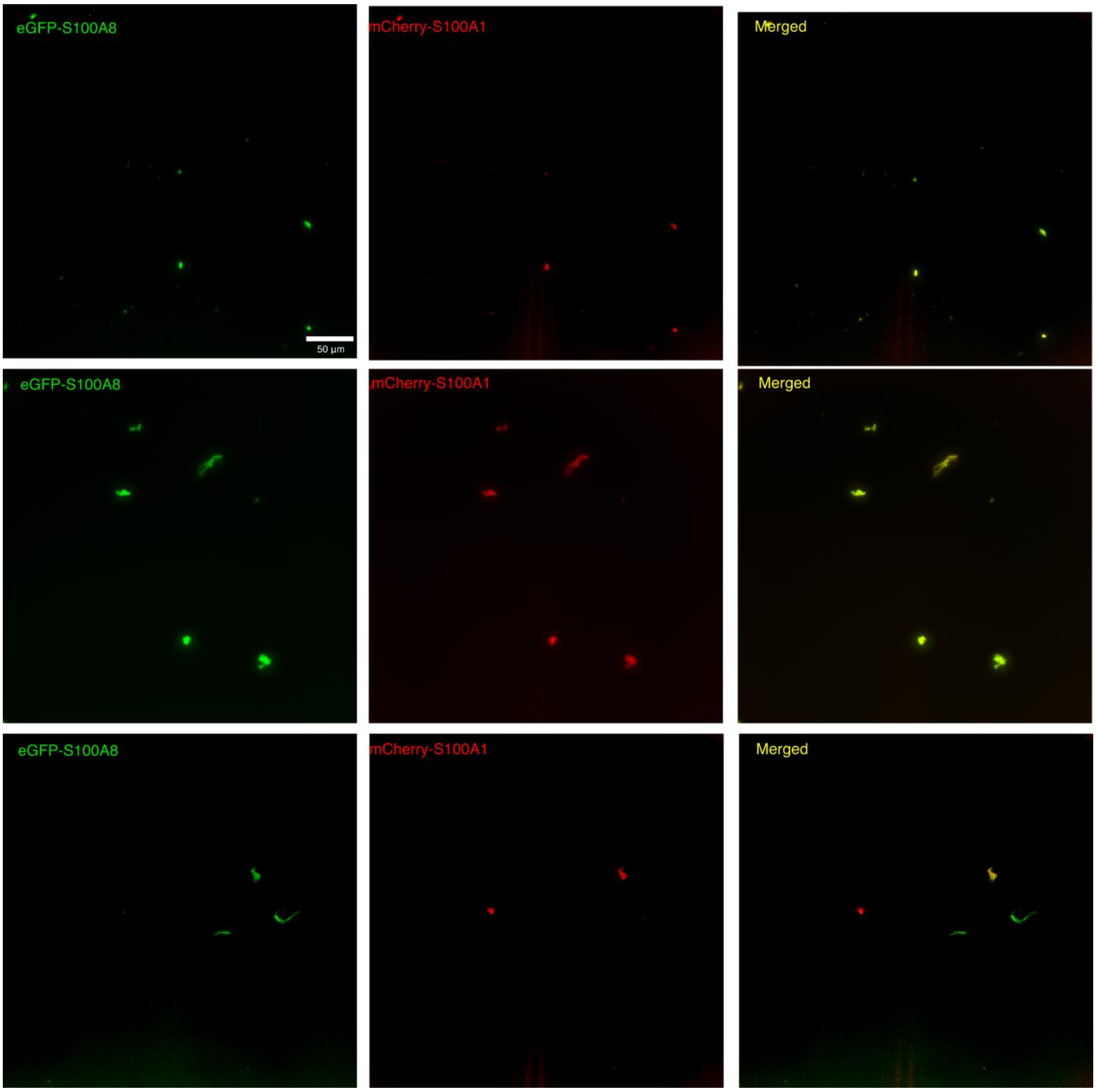

Figure S5. Fluorescence microscopy images of S100A1/S100A8 sample after aggregation in the presence of 1600 μM $CaCl_2$. Scale bar is 50 μm.

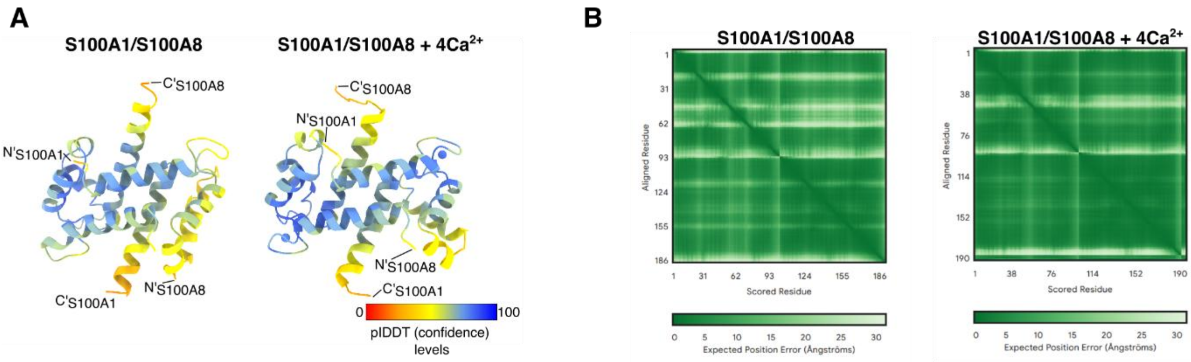

Figure S6. (**A**) Confidence levels of S100A1/S100A8 heterodimer with and without calcium ions. (**B**) 2D plots of Predicted Aligned Error, the darker green colour indicates lower position error of residue in structure.

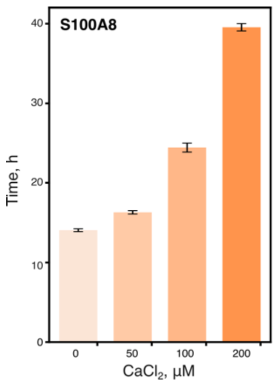

Figure S7. Inflection time of the first phase of S100A8 aggregation curves at different $CaCl_2$ concentrations.

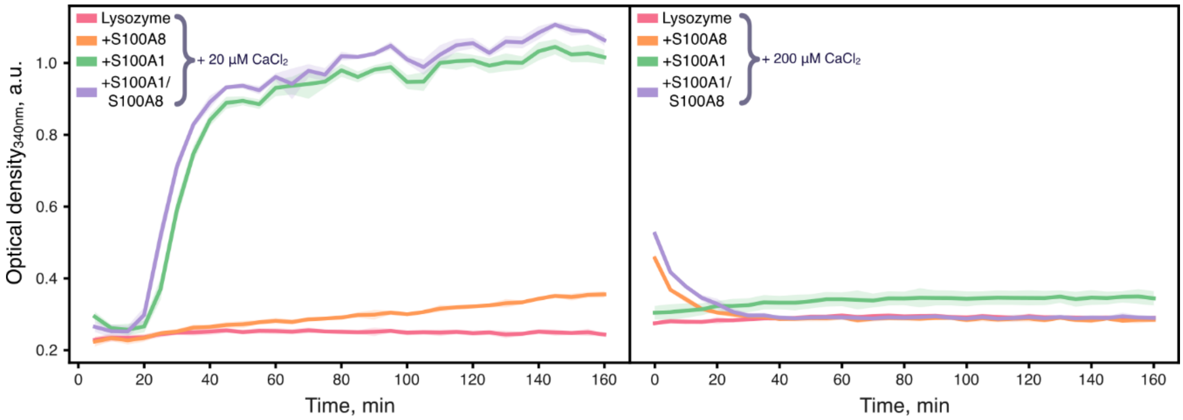

Figure S8. Chaperone activity assay of S100A1 and S100A8 (10 µM each) against DTT-induced aggregation of lysozyme (0.2 mg/ml) in the presence of 20 and 200 µM $CaCl_2$.

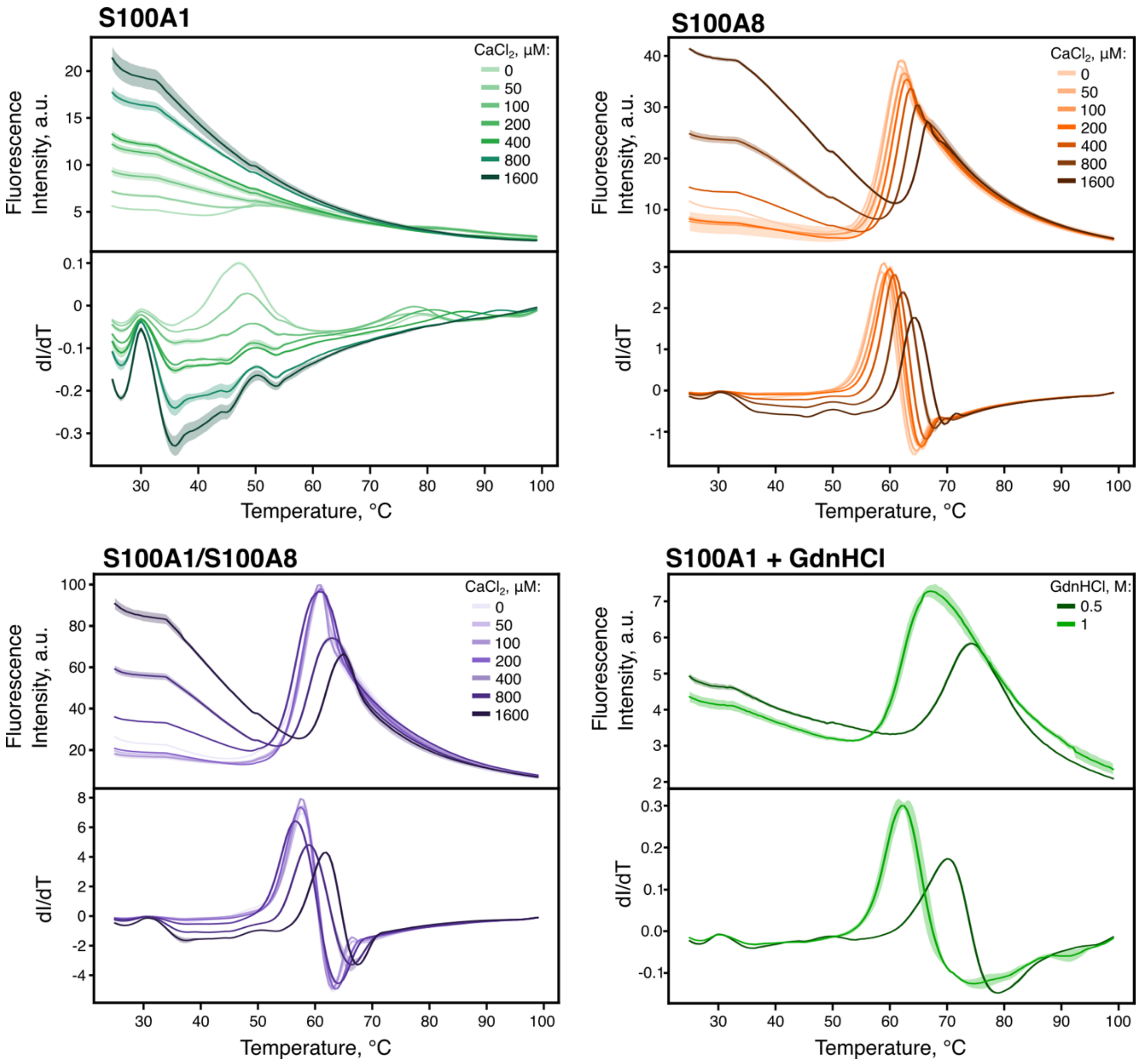

Figure S9. Thermal denaturation profiles and their first derivatives of S100A1, S100A8 and S100A1/100A8 proteins.

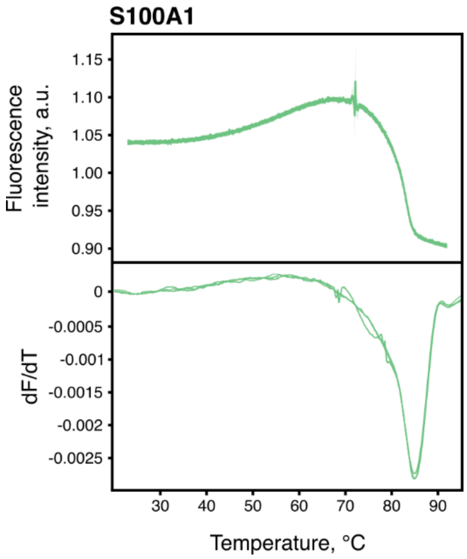

Figure S10. Thermal denaturation profiles and their first derivative of S100A1 using nanoDSF.

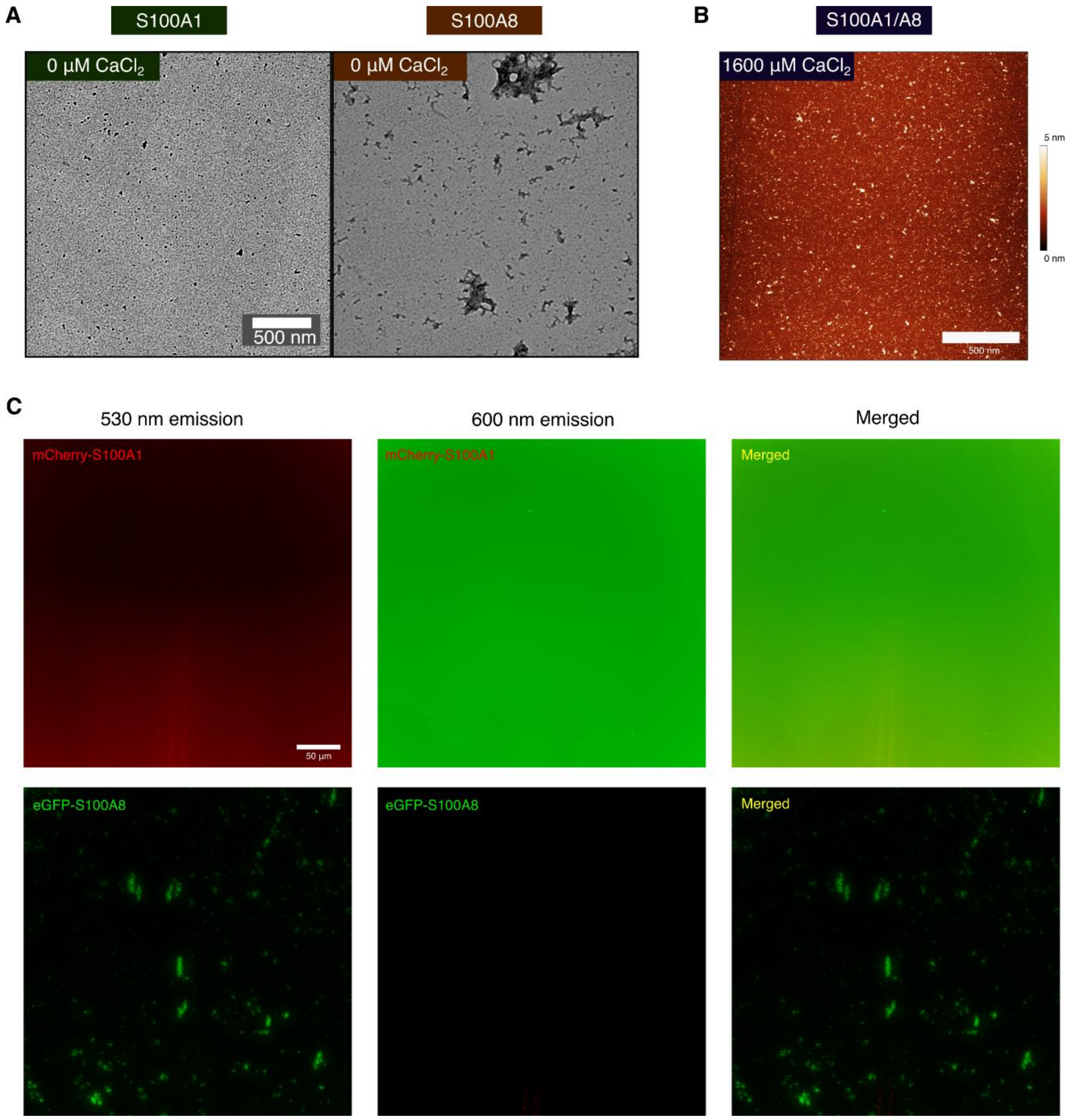

Figure S11. (A) TEM, (**B**) AFM and (**C**) fluorescence microscopy images of S100A1, S100A8, and S100A1/S100A8 samples after aggregation. Scale bars are 500 nm for TEM/AFM and 50 μm for fluorescence images. Fluorescence levels are adjusted to the same level for each protein and emission.

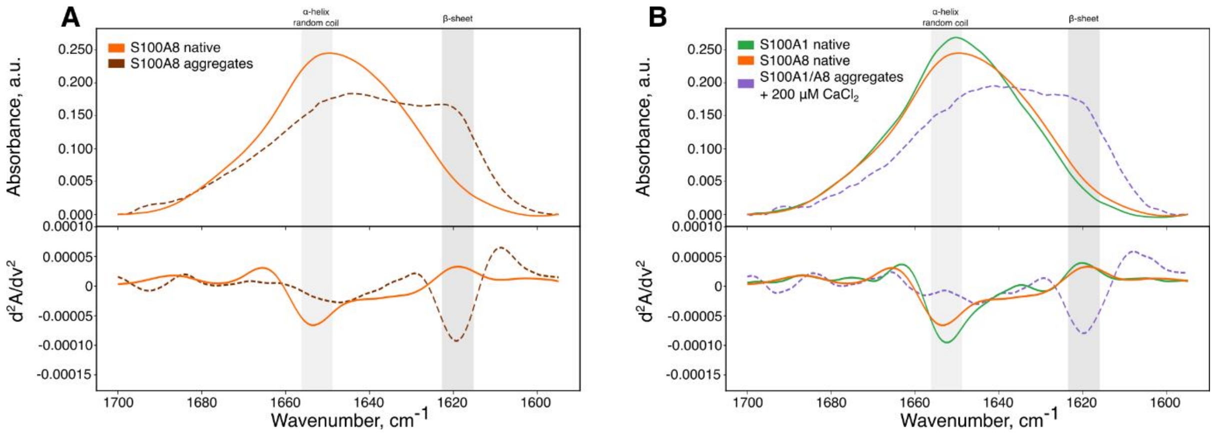

Figure S12. FTIR spectra and their second derivative of S100A8 in the absence of calcium, and S100A1/S100A8 with 200 μM $CaCl_2$, before and after 70 h of aggregation at 42 °C.

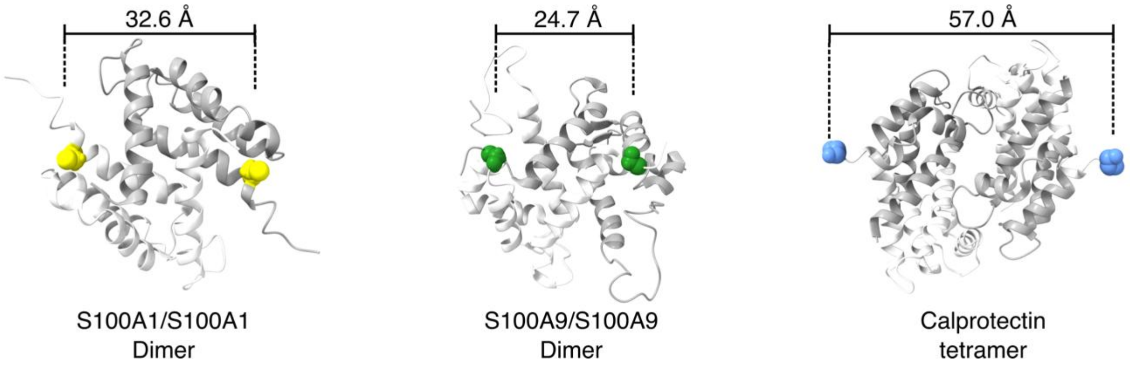

Figure S13. Measured distances between colored (spheres) residues in S100A1 dimer (PDB id: 2l0P), S100A9 dimer (PDB id: 5I8N) and tetramer structure of calprotectin (PDB id:1XK4). The colour residues correspond to the closest available positions to labelled cysteine with MTSSL.

Plasmid	Primer	Sequence
pVK1 (eGFP-S100A8)	GFP_S100A8_rf_frw	5' TGGGCCATCACCATCACCATCACGTGAGCAAGGGCGAGGAG 3'
	GFP_S100A8_rf_rev	5' CGCTTTCTCCAGCTCGGTCAACATCGAAGCTTGAGCTCGAGA 3'
pVK2 (mCherry-S100A1)	mCherry_S100A1_rf_frw	5' CCTGGTGCCGCGCGGCAGCCATATGGTGAGCAAGGGCGAA 3'
	mCherry_S100A1_rf_rev	5' TCCATAGCTGTCTCTAGTTCTGATCCCATAGAACCACCACCACCAGA 3’

Supplementary Table 1: Plasmids and respective primers used in this study.
